# Passive sampling methods for contaminated sediments: State of the science for metals

**DOI:** 10.1002/ieam.1502

**Published:** 2014-01-27

**Authors:** Willie JGM Peijnenburg, Peter R Teasdale, Danny Reible, Julie Mondon, William W Bennett, Peter GC Campbell

**Affiliations:** † Institute of Environmental Sciences (CML), University of LeidenLeiden, The Netherlands; ‡ National Institute for Public Health and the Environment, Center for Safety of Substances and ProductsBilthoven, The Netherlands; § Environmental Futures Research Institute, School of Environment, Griffith UniversityGold Coast Campus, Southport, Australia; ∥ Department of Civil and Environmental Engineering, Texas Tech UniversityLubbock, Texas, USA; # Center for Integrated Ecology, Environmental Sustainability Research Cluster, Deakin UniversityWarrnambool Campus, Warrnambool, Victoria, Australia; †† Université du Québec, Institut National de la Recherche Scientifique, Centre Eau, Terre et EnvironnementQuébec, Canada

**Keywords:** Metals, Contaminated sediment, Passive sampling, Bioaccumulation, Toxicity

## Abstract

“Dissolved” concentrations of contaminants in sediment porewater (*C*_free_) provide a more relevant exposure metric for risk assessment than do total concentrations. Passive sampling methods (PSMs) for estimating *C*_free_ offer the potential for cost-efficient and accurate in situ characterization of *C*_free_ for inorganic sediment contaminants. In contrast to the PSMs validated and applied for organic contaminants, the various passive sampling devices developed for metals, metalloids, and some nonmetals (collectively termed “metals”) have been exploited to a limited extent, despite recognized advantages that include low detection limits, detection of time-averaged trends, high spatial resolution, information about dissolved metal speciation, and the ability to capture episodic events and cyclic changes that may be missed by occasional grab sampling. We summarize the PSM approaches for assessing metal toxicity to, and bioaccumulation by, sediment-dwelling biota, including the recognized advantages and limitations of each approach, the need for standardization, and further work needed to facilitate broader acceptance and application of PSM-derived information by decision makers. *Integr Environ Assess Manag* 2014;10:179–196. © 2014 The Authors. *Integrated Environmental Assessment and Management* published by Wiley Periodicals, Inc. on behalf of SETAC.

**Key Points:**

1. Passive sampling methods (PSMs) offer the potential for cost-efficient and accurate in situ characterization of the dissolved concentrations for inorganic sediment contaminants.
2. PSMs are useful for evaluating the geochemical behavior of metals in surficial sediments, including determination of fluxes across the sediment-water interface, and post-depositional changes in metal speciation.
3. Few studies have tried to link PSM responses in sediments to metal uptake and toxicity responses in benthic organisms. There is a clear need for further studies.
4. Future PSMs could be designed to mimic saturable kinetics, which would fill the gap between the kinetic and the equilibrium regime samplers currently used, and may improve prediction of metals accumulation by benthic organisms.

## INTRODUCTION

Metals (including metalloids and some nonmetals with biogeochemical cycles that are similar to those of metals, e.g., Se) in sediments present a challenge with regard to assessing their fate and adverse effects in the environment. A basic characteristic of metals is their high reactivity and inherent tendency to bind to the diversity of ligands present within sediments. Because of fate-regulating processes such as competition, precipitation, complexation, and inclusion, and also because of the mediation of these processes by (variations in) the composition of the solid-phase matrix as well as the sediment interstitial (or “pore”) water, only a fraction of the metal present in sediments is available for transport out of the sediment and for interaction with biota (Salomons and Förstner 1984; Wang et al. 1997; NRC 2003). Therefore, such processes determine whether (competitive) interactions of metals with biota will result in adverse effects (Bryan and Langston 1992). The composition of the solid phase, in terms of sorption sites for metal binding (with clay surfaces, Al/Fe/Mn oxides, and organic coatings being especially important [Tessier et al. 1980]), and also the composition of the aqueous (porewater) phase, strongly affect the fraction of metal present that is actually available for interaction with biota. Dissolved organic carbon, pH, and major cations modify competition for abiotic and biotic ligands present in either the solid or porewater phases (Allen 2010). This bioavailable fraction is not a fixed value for a given sediment; it may well vary from one type of biological receptor to another, and it also may vary with sediment depth, depending on the oxidation/reduction potential of the system (Luoma and Rainbow 2008; Burton 2010). Typically, a thin, oxic surface layer of sediment (often less than 1 cm deep in productive sediments) overlays a suboxic zone dominated by iron(II) and manganese(II), followed by an anoxic zone where sulfide or methane is produced (Morel and Hering 1993). Note that the thickness of the oxic layer may extend to 10 cm or more in porous sandy sediments with low organic carbon concentrations. Furthermore, similar changes occur around the burrows of animals or plant roots, which can transport oxygen into the sediment. A shift from oxic to anoxic conditions dramatically changes metal speciation and can produce short-term mobilization of many metals.

Uptake of sediment-associated metals by benthic organisms can take place from the overlying water (more exactly: the benthic interfacial water, which includes burrow water and benthic boundary layer water), from porewater, and from ingested sediments (Warren et al. 1998; Luoma and Rainbow 2008). All possible uptake routes need to be taken into consideration when assessing the most relevant exposure metrics for metals, that is, water-borne and diet-borne metals. Given the fact that passive sampling methods (PSMs) respond only to aqueous metals, and given the aim of this contribution to discuss the state of the science with regard to the potential use of PSMs to evaluate metal bioavailability, the focus of this contribution is restricted to aqueous metals. Even if the route of uptake is via solids, the aqueous metal concentrations may indicate the bioavailable metals in those solids. Although the focus of the review is on benthic organisms, no attempt is made to comprehensively review all of the work that has been done on metal bioavailability in sediments.

Metal uptake from the aqueous environment can occur as a result of multiple transport mechanisms, including passive diffusion, facilitated transport, active transport, and endocytosis (Simkiss and Taylor 1995). This multitude of transport mechanisms represents a challenge for predicting metal uptake from sediments by means of PSMs. Only lipid-soluble metal species, such as neutral, nonpolar, inorganically complexed metal species, and some alkyl–metal compounds, can be taken up by organisms via passive diffusion through the cell membrane (Simkiss and Taylor 1995). Most ions are highly hydrophilic and are taken up by organisms via membrane transport proteins, such as channels, carriers, and pumps. As a result of these specific transport mechanisms, the bioaccumulation of metals may involve saturable uptake kinetics. Uptake via saturable kinetics-exhibiting mechanisms is substantially more common and toxicologically relevant than passive diffusion or active transport, although active transport can also be saturable (Simkiss and Taylor 1989; Wood 2001). Once the metal is taken up, it is subject to internal processing, can be shuttled to many subcellular fractions, and can react within the organism in a variety of ways that allow for continued transport; thus, the establishment of true equilibrium, in general, is unlikely for ionic species.

Organisms have developed physiological or anatomical mechanisms to adapt to metal exposure (Fairbrother et al. 2007). They are often able to actively regulate metal bioaccumulation and maintain homeostasis over a range of exposures via either exclusion or increased elimination (Wood 2001; Rainbow 2007). Additionally, organisms have the ability to regulate internal metal concentrations through sequestration, detoxification, and storage (Mason and Jenkins 1995; Rainbow 2002; Vijver et al. 2004). Because of the natural occurrence of metals in the environment, these physiological processes have evolved over time, allowing organisms to adapt to excess metals and to accumulate essential metals at required levels. In contrast, uptake by PSMs is not complicated by such biological processes and is determined simply by mass transfer principles.

Consequently, traditional measures of metal bioavailability, that is, the concentration taken up by the organism, do not necessarily translate into toxicological bioavailability, because this metric may not reflect the metal that actually reaches the site of toxic action. Metal toxicity depends on the chemical form or compound, interactions between metals, and the binding affinity of a specific receptor site (USEPA 2007). Toxicity to organisms occurs when they cannot deal with the overwhelming flux of metals to specific internal receptor sites, which may come from both water-borne and diet-borne metals. In addition, the effective toxicological “dose” of metals and metal complexes is often life stage- and gender-specific. Uptake and organ distribution kinetics are also often species-specific. Thus, tissue-specific bioaccumulation does not necessarily equate to adverse effects but rather depends on the toxicokinetics of bioreactive forms of metals in competition for enzymes and binding sites associated with metal pools that are not metabolically active (Hook and Fisher 2002).

Two distinctly different approaches have been used to predict metal bioavailability in aquatic environments: equilibrium modeling (e.g., the Biotic Ligand Model or BLM—Di Toro et al. 2001; Campbell and Fortin 2013;) and biodynamic modeling (e.g., the DYNBAM model—Luoma and Rainbow 2005; Rainbow 2007). The BLM aims to predict how dissolved metals interact with, and eventually affect, aquatic organisms. The epithelial surface of the organism, including the so-called “biotic ligand,” is assumed to be in equilibrium with the ambient water, and the biological response (metal uptake and/or toxicity) is predicted on the basis of the concentration of the metal–biotic ligand complex, metal–biotic ligand–epithelium. An implicit corollary of the BLM approach is that metal toxicity can be predicted on the basis of water-borne exposures and that metals in food can be ignored. In contrast, the biodynamic approach considers uptake of metals from both the ambient water and the ingested solids, and also takes into account metal loss (excretion, elimination of fecal matter). Considering both water and food as vectors would be preferable when assessing the bioavailability of metals in sediments. However, as indicated previously, in the present case we are addressing the potential use of PSMs to evaluate metal bioavailability; because PSMs only respond to aqueous metals, we have focused on the sediment porewater and the equilibrium modeling approach.

**Figure 1:**
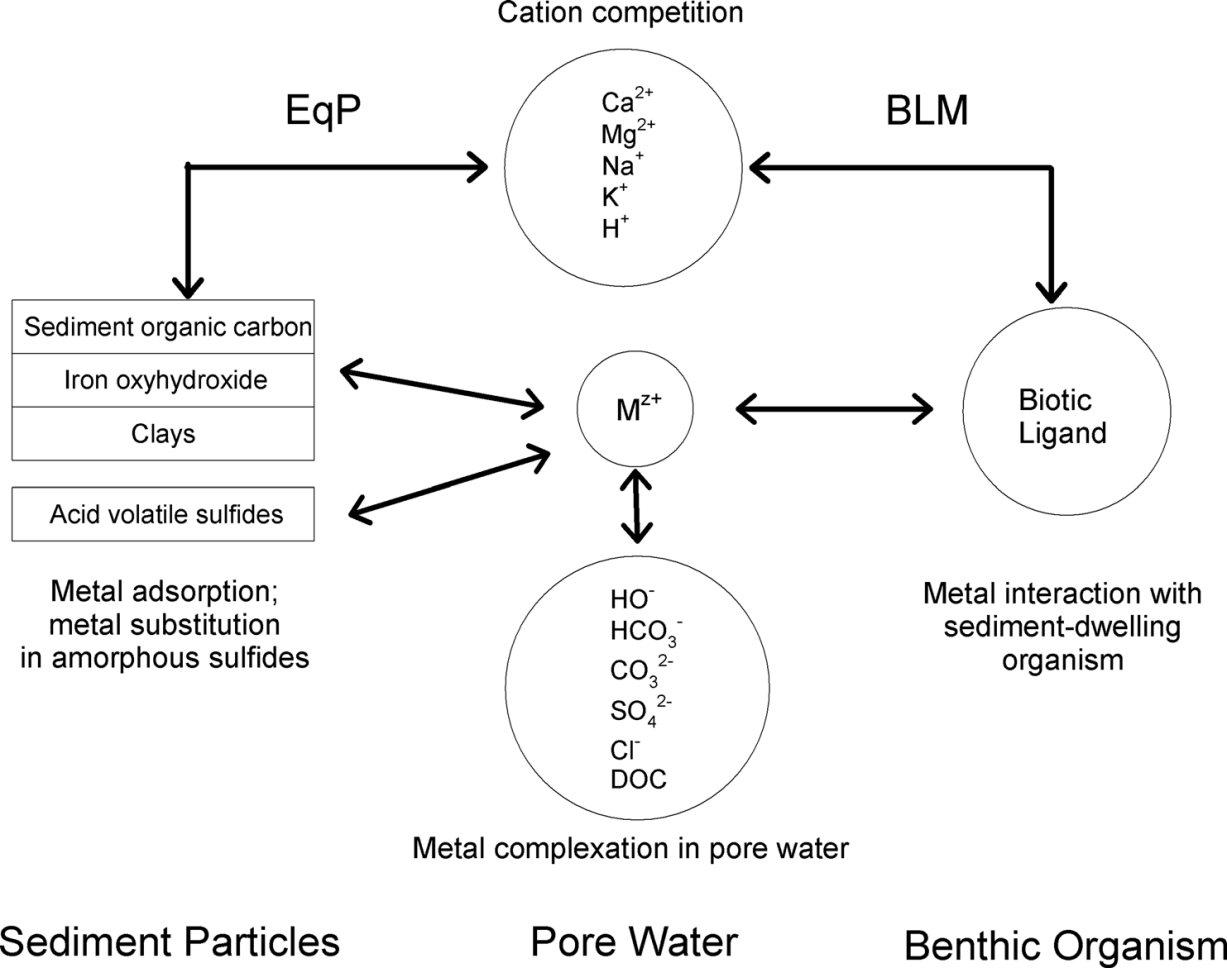
Schematic representation of processes governing metal bioavailability in sediment porewater (modified from Di Toro et al. 2005). M^z+^ corresponds to the free metal ion or C_free_; BLM = Biotic Ligand Model.

Equilibrium partitioning models have been used to predict dissolved concentrations in porewater for selected metals (USEPA 2005). The emphasis on dissolved metals in the porewater derives from 2 considerations. First, contaminants in sediment porewater (*C*_free_) integrate the numerous interactions that exist between the dissolved metal and the various solid phases present in the sediment, and thus such measurements may provide an estimate of the overall activity of the metal in the sediment environment (Ankley et al. 1994); the bioavailability of the metal may be directly related to its chemical activity. Second, extensive literature demonstrates that the bioavailability of dissolved metals to aquatic organisms normally varies as a function of the free metal ion concentration in solution [M^z+^] (Campbell 1995), and that this interaction of the organism with [M^z+^] is modulated by pH and water hardness ([Ca^2+^], [Mg^2+^]) (Di Toro et al. 2001). Although the importance of the free metal ion was initially recognized for aquatic organisms living in the water column, it has recently been extended to benthic organisms and the sediment environment, again with an emphasis on porewater (see Di Toro et al. 2005 for a description of this “sediment biotic ligand model”). Whereas debate continues about which solid phases control porewater metal concentrations (e.g., acid-volatile sulfide [AVS]; amorphous iron oxyhydroxides; solid organic matter) (Nguyen et al. 2011), *C*_free_ offers a very useful “window” into understanding metal bioavailability in sediments. In principle, the emphasis should be on the free metal ion concentration in the sediment porewater (*C*_free_), not the total dissolved metal concentration. If the PSM provides estimates of the total dissolved metal concentration, as well as the other water relevant chemistry parameters (e.g., pH, dissolved organic carbon, hardness, alkalinity), *C*_free_ can be calculated using appropriate equilibrium models (Milne et al. 2003; Tipping et al. 2011). The processes underlying the current concepts for assessment of metal bioavailability in sediments are schematically depicted in Figure 1.

The porewater approach strictly only applies for selected cationic metals in which uptake is dictated by exposure from the sediment porewater, for example, by rooted aquatic plants or by benthic animals with metal-permeable epithelia. Provided the physical and chemical conditions in the digestive tract do not differ markedly from those in the host sediment, the relationship between porewater metal concentrations and metal toxicity should still hold even if uptake from ingested metals (i.e., from the sediment solid phase) occurs. If, however, the conditions in the digestive tract differ markedly from those in the adjacent sediment, particularly with respect to lower pH, and if the transit time in the gut for the sediment is fairly long, the relationship between [M^z+^]_interstitial_ and metal bioavailability may not apply (Campbell et al. 2005).

Although normalization of the total concentrations in the sediment with respect to the concentration of the dominant binding phase (e.g., AVS for divalent metals) often works well for laboratory-spiked sediments, this approach does not reliably characterize the bioavailability of contaminants in field sediments. This is because of the presence of other binding phases (e.g., sedimentary organic carbon or iron oxyhydroxides) and the heterogeneity of metal-sequestration and mobilization processes (Campbell and Tessier 1996; Jonker et al. 2004; Nguyen et al. 2011).

Given the cost implications of remedial decisions, these findings have led to a growing body of literature on the use of PSMs and partitioning-based approaches for direct analytical quantification of the bioavailability of sediment-associated contaminants (Maruya et al. 2011). Past research has focused heavily on organic compounds, but a growing body of literature reports on the use of PSMs for assessing metal availability (Davison et al. 1994; Martin 2008).

We provide an overview of the approaches taken to assess metal bioavailability by means of PSMs, focusing on bioaccumulation and toxicity in sediments and overlying waters. We conclude with a summary of knowledge gaps and the next logical steps to be taken to fill those gaps.

## PSMS FOR METALS

Despite significant advances in the development and application of PSMs, practical incorporation of these relatively simple, sensitive methods in contaminated sediment management decisions has been limited. Barriers to broader regulatory acceptance and use are a lack of understanding of the advantages and limitations of PSMs over traditional analytical methods; confusion with regard to the different PSM materials and configurations that are increasingly reported in the literature; lack of consensus on technical guidance, and, as we see in this review, the paucity of studies that relate measurements with PSMs to metal uptake and toxicity in benthic organisms. Examples and key details of PSMs used to measure metals are compiled in Table 1, including their initial use for water monitoring, if relevant. The 4 main types of PSMs used to investigate metals in sediment are summarized later. The equilibrium-regimen and kinetic-regimen operation of PSMs are described thoroughly elsewhere (Lydy et al. this issue).

**Table 1:** Overview of PSMs for metals

A - Overlying Water		
Technique & metal	Description and operation	References (examples)
• Diffusion samplers (trace metals and major ions)	Simple diffusion cells are left in the water column to equilibrate with the ambient water. Equilibration time is dependent on the depth or design factor (F, cm) of the sampler device, with some samplers equilibrating in about 24 h in well-mixed waters (F = 1.0 cm) but others requiring 2 or more days.	(Benes and Steinnes 1974; Davison et al. 2000; Fortin et al. 2010)
• Diffusive gradients in a thin film (DGT) samplers (Al, As, Cd, Cu, Se, Hg, Ni, Pb, U, Zn; lanthanides)	Diffusion gradients in thin films give an estimate of “labile” metals, which are operationally defined by the nature and thickness of the gel through which the metals must diffuse, and by the strength of the binding phase. Kinetic regimen sampler, normally deployed from several hours to days. Use of a diffusion coefficient assumes no calibration is required.	(Davison and Zhang 2012; Dunn et al. 2003; Garmo et al. 2003; Zhang and Davison 1995)
• “Chemcatcher” samplers (Cd, Cu, Ni, Pb, Zn, Hg)	Teflon watertight body that retains a chelating disk; receiving phase is overlaid with a cellulose acetate diffusion-limiting membrane (0.45 µm pore size; 0.135 mm thick). Kinetic regimen sampler, deployed for periods of 1 to 4 weeks; requires laboratory calibration	(Aguilar-Martinez et al. 2011; Allan et al. 2008)
• “Gellyfish” samplers (Cu)	Polyacrylamide gel, embedded with iminodiacetate (Chelex) resin particles, is suspended in water until equilibrium reached; the amount of metal in the resin is proportional to the free metal ion concentration in the water. This technique has only been used in coastal marine systems. Deployment times of at least 8 d are needed to achieve equilibrium.	(Senn et al. 2004)
• Hollow fiber supported liquid membranes (Cd, Cu, Mn, Ni, Pb, Zn)	A hollow fiber is impregnated with a crown ether (ligand), dissolved in an organic solvent; the metal–ligand complex migrates across the fiber membrane and the metal is released to the aqueous acceptor phase in the lumen of the fiber. Can be used as either an equilibrium or kinetic passive sampler and provides the best estimation of free metal ions	(Bautista-Flores et al. 2010; Bayen et al. 2007; Parthasarathy et al. 1997; Sigg et al. 2006; Slaveykova et al. 2009)

B - Sediments		
• Porewater peepers (Al, As, Cd, Co, Cu, Hg, Fe, Mn, Ni, Pb, Zn)	Multilayer diffusion cells inserted into sediments and allowed to equilibrate with the sediment pore water. Equilibration times range from about 1 day to well over 1 month (F = 1.0 cm), depending on whether the resupply of analytes from the sediment solid phase is able to sustain the pore water concentrations. The equilibration process has been modeled. Typical deployments of about 1–2 weeks are used.	(Brandl and Hanselmann 1991; Carignan et al. 1985; Hesslein 1976; Nipper et al. 2003; Teasdale et al. 2003)
• Teflon sheets (Fe, Mn + associated metals)	Teflon sheets act as a “substrate” for the deposition of iron and manganese oxyhydroxides and their associated metals; useful for identifying the boundary between the oxic and sub-oxic zones and adsorption of metals to new phases.	(Belzile et al. 1989; Feyte et al. 2010; Fortin et al. 1993)
• DGT samplers (As, Cd, Cu, Co, Hg, Ni, Pb, V, Zn)	DGT deployments within sediments are influenced by sediment response to depletion of analytes in the pore waters. The mobilization of metals from sediment solid phase can lead to pore water concentrations being fully or partially sustained. The response of sediment to DGT measurements has been characterized by several models, including interpretation of micro-niche responses. Metal pore water distributions can be determined at high resolution and in 2 dimensions.	(Davison et al. 2000; Davison et al. 1997; Stahl et al. 2012; Zhang et al. 1995)
• Diffusive equilibration in a thin film (DET) samplers (Cd, Cr, Cu, Fe, Mn, Mo, Pb, Re, U, Zn)	An equilibrium passive sampler that uses a thin layer of hydrogel as the sampling media. Deployed in unconstrained or constrained modes, with the former requiring fixing to minimize diffusional relaxation and the latter using compartments. Can be used to make measurements of 2-dimensional pore water distributions at high resolution.	(Davison and Zhang 1994; Fones et al. 2001; Krom et al. 1994; Yu et al. 2000)

Sediment deployments require careful consideration of issues such as how to deploy PSMs without disturbing the sediment chemistry, or how to ensure the samples are adequately preserved, especially when the sampled sediment includes suboxic and anoxic zones. Sediment chemistry can most readily be disturbed by introduction of oxygen into the sediment or the sample collection matrix. To prevent this, the assembled PSMs are thoroughly deoxygenated before being deployed (Carignan et al. 1994). The PSMs are usually deployed upright so that vertical profiles or distributions are obtained. Divers will be needed in deep waters, but benthic landers have also been used (Fones et al. 2001). In either instance, if care is taken during the PSM deployment, so that the sediment is minimally disturbed and good contact occurs between the sediment and the PSM sampling surface, then sampling artifacts can be minimized relative to the period over which diffusive sampling occurs. When the PSM device is retrieved, subsampling or preservation needs to be done quickly to minimize the effects of oxygen exposure. Some PSMs also can experience diffusional relaxation and need to be either sub-sampled or quantified within a short period. Other issues include making certain that equilibrium-regimen PSMs have approached equilibration and interpreting the sediment response to kinetic PSMs. Fortunately, various models have been described to assist with these issues. Issues specific to each type of PSM are discussed later, along with the theory relevant to each PSM technique. The Supplemental Data contains case studies on the application of PSMs for investigation of metals and metalloids in sediments.

### Porewater peepers

Peepers were first described in 1976 by Hesslein (1976) and were developed for use with metals by Carignan and co-workers (1985). They are equilibrium regimen PSMs, in which solutes from adjacent porewaters diffuse across a membrane into compartments containing water. Porewater peepers exist in several different designs, but they often consist of an acrylic (Plexiglas) frame, into which have been milled a series of sampling wells (Carignan et al. 1985; Hesslein 1976). The sampling wells are filled with ultrapure water, covered with a precleaned microbially resistant membrane (usually polysulfone, 0.2 µm nominal pore size) that is secured in place with a cover plate (Supplementary Data Figure S1). In addition to using trace-metal clean sampling practices (Nriagu et al. 1993), the entire peeper assembly must be deoxygenated under a nitrogen atmosphere before deployment (Carignan et al. 1994), otherwise the oxygen held within the plastic of the frame can diffuse into the (anoxic) water in the sampling cells located below the redox boundary and alter the chemistry of the collected porewater.

Properly prepared peepers are carefully inserted into the sediments of interest and left in place for days to weeks, until chemical equilibrium is approached. The crucial parameter for peepers is the design factor (F, cm), which is the effective depth of the peeper cells (derived from the cell volume/exposed membrane surface area; Brandl and Hanselmann 1991). The larger the design factor, the longer the equilibrium time; peepers typically have F values of 0.5 cm or greater. This is discussed further in the section on use of models.

Multiple peepers are typically deployed at a given sediment site to account for sediment heterogeneity. The peeper is inserted vertically into the sediment, leaving several wells exposed above the sediment–water interface to sample both the interstitial and overlying water. When the peepers are retrieved and quickly cleaned, one must note closely where the sediment–water interface was situated and then subsample the individual sampling wells without delay (minutes), starting with the bottom wells that presumably contain anoxic samples (Teasdale et al. 2003). The water collected from the peeper wells is normally used to measure the total dissolved concentrations of the various solutes. A potential advantage of using peepers is that these dissolved concentrations can be compared directly with water quality criteria, which are widely used in risk assessment and for which a large body of toxicological data exists.

**Figure 2:**
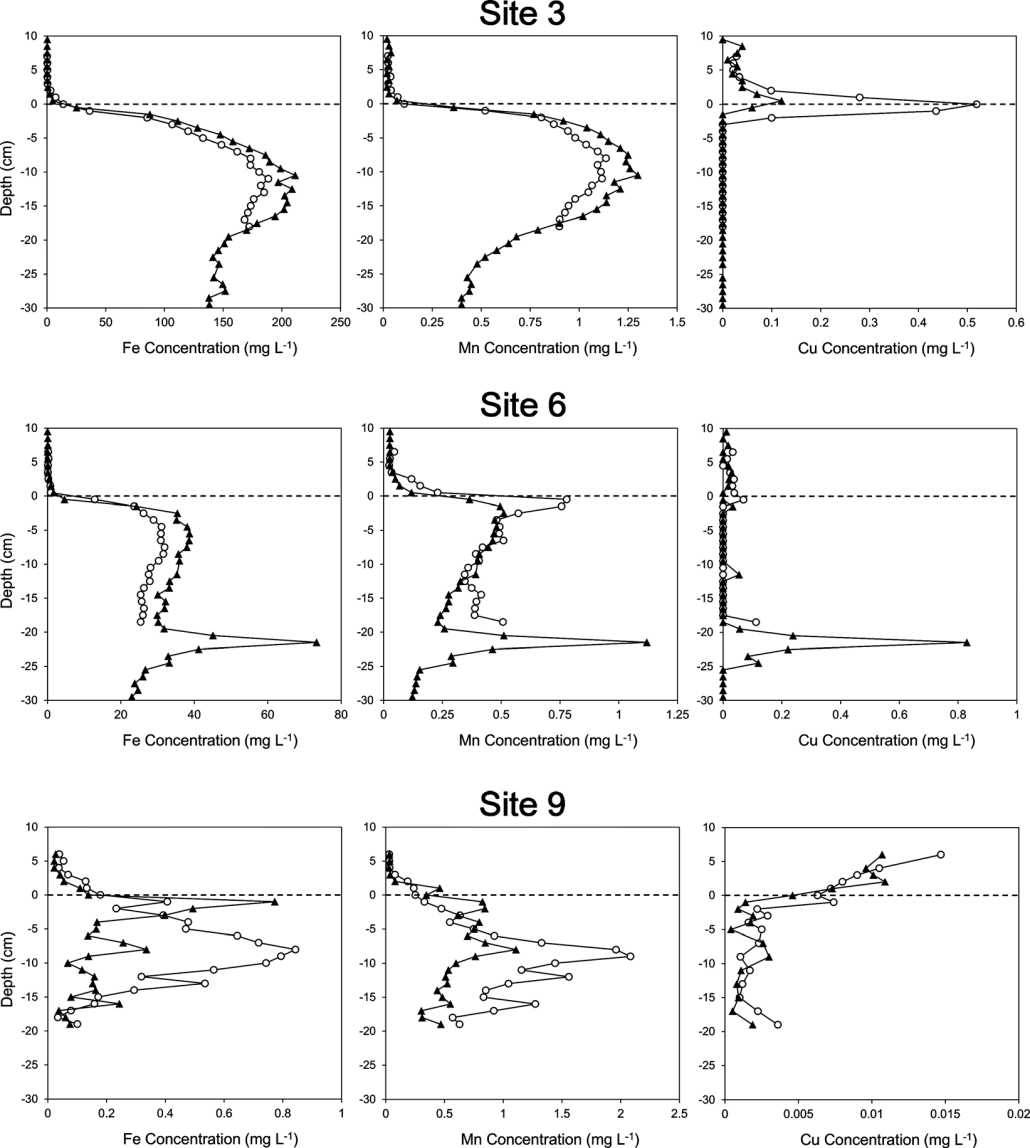
Typical profiles of Fe, Mn, and Cu as observed at 3 sites in a Cu-contaminated Australian harbor (P. Teasdale, Griffith University, Gold Coast Campus, Australia, personal communication); ppm = mg · L^−1^. Double-sided peepers (F = 0.5 cm) were deployed for 5–6 days.

Samples obtained from the peeper wells are preserved in the same way that a surface water sample would be treated, that is, without trying to maintain the redox state of the original sample. For example, subsamples destined for metal analysis are normally acidified with trace-metal grade HNO_3_; a typical subsampling and preservation protocol was described by Alfaro-De la Torre and Tessier (2002). Typical porewater profiles obtained with peepers are shown in Figure 2 for Fe, Mn, and Cu from 3 sites in Macquarie Harbour, Australia. Peepers X, D, N, and V had 26 chambers at 1-cm resolution, and peepers U and T were a meter long, although porewater profiles are only shown to 30-cm depth. Site 3 was an artificial delta comprising tailings from a Cu mine operating continuously for more than 100 years. Site 5 was approximately 3 km down-gradient of site 3. Site 9 is 30 km from the delta at the south end of the harbor, where strongly sulfidic sediment is influenced by tannin-rich inflow. The porewater profiles of Fe, Mn, and Cu clearly reflect changes in sediment conditions and extent of contamination.

### Diffusive equilibration in thin films

The diffusive equilibration in thin films (DET) technique, first described by Davison et al. (1991), is also an equilibrium regimen PSM. For DET samplers, a thin hydrogel layer (usually less than 1 mm thick) is used as the sampling medium instead of water; the F value for DET devices is equal to the gel thickness (<0.1 cm). This means that DET samplers equilibrate much more quickly than peepers and also allow measurements at higher resolution (the measurement resolution is limited by the gel thickness, which determines the possible extent of lateral diffusion). Two main types of DET devices are used. Unconstrained DETs use continuous sheets of hydrogel, and porewater profiles can undergo diffusional relaxation when they are removed from the sediment. They are normally preserved by soaking immediately in NaOH, which causes iron and manganese hydr(oxides) to precipitate and solutes to adsorb to these newly formed phases (Davison et al. 1991). Alternatively, subsamples can be collected immediately, by slicing the gel at a particular resolution (e.g., 1–3 mm); subsamples are then eluted (in 1 M HNO_3_ or NaOH) and measured separately (Mortimer et al. 1998; Pagès et al. 2012). A recently developed approach used for 2-dimensional measurements by DET has been to expose the retrieved hydrogel to colorimetric reagents soon after removal, followed by quantification on-site by computer imaging densitometry (see section on *Two-dimensional measurements of metal in sediment*). The second type of DET is a constrained probe in which agarose hydrogel is added to compartments that are not in diffusional contact with each other and that can be removed, under clean laboratory conditions, eluted (as described previously) and measured separately (Fones et al. 1998).

Because DET samplers do not concentrate analytes and usually require an elution step, DET has been used infrequently for trace metal determination (Yu et al. 2000) and is much more commonly used for Fe or Mn (Fones et al. 2001; Bennett, Teasdale, Welsh, Panther, Jolley et al. 2012). A recent interest in DET for metals in sediments is with the development of high-resolution, 2-dimensional measurements of porewater iron(II) (Shuttleworth et al. 1999; Pagès et al. 2011) and other analytes (see the next section on *Two-dimensional measurements of metals in sediment*). These approaches can be combined with other PSM measurements of metals (especially DGT measurements) to allow the major biogeochemical processes and sediment heterogeneity to be characterized, which can assist with interpretation of the major processes influencing metal mobilization.

### Diffusive gradients in thin films

The diffusive gradients in thin films (DGT) technique, first described by Davison and Zhang (1994) for the analysis of Zn in seawater, is a kinetic regimen passive sampler. Diffusive gradients in thin films technique uses a thin hydrogel layer (as used for DET) to constrain mass transport of solutes by diffusion into a binding layer containing a resin or functional groups that will selectively bind or adsorb the analyte species of interest. As with all kinetic regimen PSMs, a linear relationship exists between the accumulation of analytes and deployment time for measurements that do not approach the capacity of the binding layer and do not deplete the analyte concentration in the surrounding media.

Most studies on developing and applying PSMs for metals have focused on the DGT device. DGT is a well-developed technique for sampling metals in bulk water, with deployments from 6 to 72 h being typical. In the well-mixed overlying waters, the water motion maintains an essentially constant pool of analyte, and the mass transfer resistances external to the device are often minimal, leaving the rate of metal uptake by the resin gel controlled by the diffusive layer. The rate of diffusion in this layer (the diffusion coefficient) is similar to that of the diffusion rate of the metal ion in pure water and can be measured accurately by experiment (Zhang and Davison 1999). Diffusion coefficients for many metals in polyacrylamide gels are available in the DGT literature, often provided along with newly described DGT methods (Garmo et al. 2003; Scally et al. 2006; Panther et al. 2013). The analyte concentration in the water adjacent to the sampler is related to mass adsorbed in a given time and can be calculated using the DGT equation:
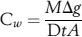
where

*M* = mass of metal adsorbed to the binding layer (ng)
*C*_*w*_ = concentration of metal in bulk solution (ng · mL^−1^)
D = diffusion coefficient of metal in gel (cm^2 ^· s^−1^)
*t* = deployment time of the device (s)
*A* = exposure area of the device to the bulk solution (cm^2^)
*Δg *= distance from the resin layer to the bulk solution (cm) (Zhang and Davison 1995)

The mass in the binding layer is determined by conventional analytical techniques, after an elution step in which an elution efficiency correction is used to estimate the actual accumulated mass.

The components of a DGT water sampler are shown in Supplemental Data Figure S2, along with a representation of how the diffusive gradients develop. The diffusive and resin gel layers originally designed by Davison and Zhang were synthesized from an acrylamide and agarose cross-linker solution with an ammonium persulfate initiator and an N,N,N',N'-tetramethylethylendiamine catalyst (Zhang and Davison 1995). The binding layer includes a specific adsorbent chosen for the specific metal of interest, typically Chelex®-100 resin for common divalent metal ions. The layers are manufactured by mixing the aforementioned constituents and casting to a desired thickness between 2 glass plates (Zhang and Davison 1995). Finally, a filter is placed on top of the diffusive layer facing outward; this filter is normally a 0.45-µm cellulose nitrate membrane that serves to prevent particles from binding to the outside of the diffusive layer and penetrating into the gel layers (Zhang and Davison 1995), although in sediment deployments a polysulfone membrane is preferred. Subsequent research has expanded the number of analytes measurable by DGT, by modifying the adsorbent within the binding layer, to include many important anionic species such as As, Se, V, Mo, Sb, and W (Table 2). The extraction solutions used with each binding layer can vary considerably, and this is a major step in the development of a new DGT technique. The measurement of Hg and As species is described in more detail in the Supplemental Data.

**Table 2:** Analytes measurable with common DGT binding phases

DGT Binding Phase	Measurable Analytes	Reference(s)
Chelex 100	Al^a^, Cr, Mn, Fe^b^, Co, Ni, Cu, Zn, Ga, Cd, Pb, La, Ce, Pr, Nd, Sm, Eu, Gd, Tb, Dy, Ho, Er, Tm, Tb, Lu, Y	(Garmo et al. 2003; Warnken et al. 2004b; Zhang and Davison 1995)
Ferrihydrite^c^	V(V), As(III), As(V), Se(VI), Mo(VI), Sb(V), W(VI), U	(Luo et al. 2010; Stockdale et al. 2010)
Metsorb (TiO_2_)^d^	Al, V(V), As(III), As(V), Se(IV), Mo(VI), Sb(V), W(VI), U	(Bennett et al. 2010; Hutchins et al. 2012; Panther et al. 2012; Panther et al. 2013)
Mercapto-silica	As(III) selectively, Hg	(Bennett et al. 2011; Fernández-Gómez et al. 2011)

a Only suitable for acidic freshwaters (Panther et al. 2012).

b Requires further method validation to ensure accurate results under all deployment conditions (Garmo et al. 2003).

c Not suitable for marine waters (Bennett et al. 2011; Panther et al. 2013).

d Suitable for fresh and marine waters, except Mo and U, which are only measurable in freshwaters (Hutchins et al. 2012; Panther et al. 2013).

Although simple in concept, the interpretation of DGT results can be difficult because of the potential for species and complexes operationally defined as “labile” to be sorbed onto the resin gel (labile = free ions and inorganic and organic complexes that dissociate over the time it takes to diffuse into the device or within the sampling period). The mass of analyte accumulated onto the resin gel depends on the relative diffusion rates of species that may pass through the gel layer. A “restricted” diffusive gel layer, with bis-acrylamide cross-linker, in which the average pore size is smaller than in the normal diffusive gel, can be used to substantially decrease the contribution to the measurement of organic metal complexes (Zhang and Davison 2000).

Several issues can occur with DGT measurements in the water column. In poorly mixed solutions a diffusion boundary layer of a significant thickness will develop, which effectively increases the thickness of the diffusive layer and thus decreases the flux of metal to the sampler. This is easily overcome, however, by using multiple samplers with differing diffusive layer thicknesses, which allow the accurate calculation of the diffusion boundary layer thickness (Zhang et al. 1998; Warnken et al. 2006; Bennett et al. 2010). Biofilms can form on the sampler surface during longer deployment times and can be a significant problem for water-column DGT measurements under some circumstances (Uher et al. 2012). The DGT measurement is therefore considered highly operational; the measured concentration is the effective free metal ion concentration (attributable to use of the diffusion coefficient of free ions) that would be required to account for the accumulated mass over the deployment period, but it is based on well-understood chemical and physical processes.

The DGT devices have also been thoroughly characterized for use in sediments, for which different configurations of PSM devices are used (Supplementary Data Figure S3), because of an interest in concentration profiles or distributions. For sediments in which porewater solutes are fully sustained by rapid desorption from the solid phase, concentrations can be calculated using the DGT equation described previously. The DGT devices can be placed in the sediments for varying periods, from 6 to 8 h to over a week, although deployments of 1 to 3 days are more common. In many sediments, however, the available pool of metals that can be taken up by the binding gel may become limited because of their depletion in the porewaters and limited release from the sediment or slow diffusion or convection of analyte solutes to the DGT sampler. This depletion becomes more significant over longer deployment times. The net effect is that the accumulated mass is often less in sediments than in water at the same concentration. For this reason, laboratory work is typically needed to identify any potential matrix effects, to confirm the species that are measurable as well as the efficiency of measurement, and to allow optimization of the sampler binding layers, diffusive gel layers, and other operational parameters, such as deployment time. Laboratory experiments also can be used to validate the DGT technique by comparing porewater DGT measurements with another measurement of porewater solute concentration.

**Figure 3:**
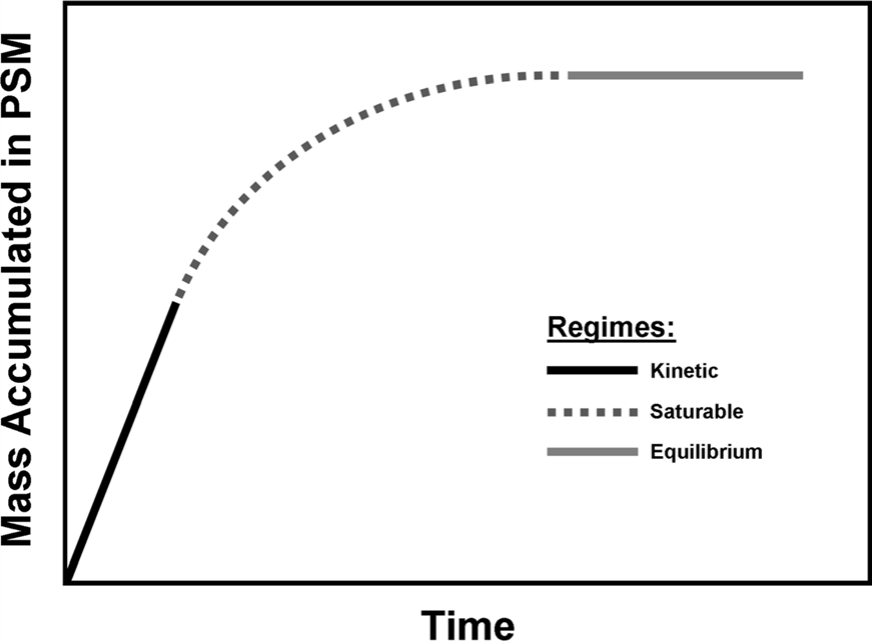
Illustration of the gap to be filled between the current generations of kinetic and equilibrium regimens traditionally used by PSMs.

Finally, as discussed later, PSMs can better characterize fine-scale changes across the sediment–water interface, or with respect to other sediment biogeochemical zones or features, than conventional measurements. New binding layers could be used that have lower binding capacities and selectivity for metals that may better mimic the saturable kinetics often observed in bio-uptake and with related biotic ligands that are the sites of toxic action (i.e., between the kinetic and equilibrium regimens traditionally used by PSMs), as illustrated in Figure 3.

### Teflon sheets

The foregoing PSM techniques have focused on the sediment porewater rather than the various solid phases with which the porewater is in equilibrium. Direct determinations of metal–solid associations in aquatic sediments, for example, by X-ray diffraction, have rarely proved feasible. The great variety of metal forms in sediments, the presence of complex organic–mineral associations, the amorphous nature of the most important metal-binding phases (e.g., Fe/Mn oxyhydroxides), and the relatively low metal concentrations involved have all combined to limit this approach. However, Tessier and co-workers devised a method to collect authigenic metal-binding phases directly, on small Teflon® sheets (Belzile et al. 1989; Fortin et al. 1993). When such sheets are inserted vertically into lake sediments and left there (for months to years), authigenic Fe and Mn oxides and their associated material are deposited in distinct bands at or above the oxic–anoxic interface; the iron oxyhydroxides appear as a thin firmly fixed orange-brown band (typically ∼0.5 cm wide and 5–20 µm thick), whereas the manganese oxyhydroxides occur as a brownish, more diffuse layer located above the Fe deposit. In some cases, a band or discrete spots corresponding to amorphous sulfides also form below the Fe deposit.

The Teflon sheets are inserted into the sediments and retrieved manually, normally by divers. On retrieval, the Teflon support is rinsed in ambient lake water, and the bands corresponding to the authigenic metal-binding phases can then be characterized directly (e.g., X-ray diffraction; transmission electron microscopy) and analyzed for their elemental content. The amounts of collected material are small (e.g., ∼500 µg of Fe oxyhydroxide per sheet [Tessier et al. 1996]), but the samples collected in this way are relatively free from contamination by the other solid phases present in the sediment matrix. Recent applications of this technique include biogeochemical investigations of As (Couture et al. 2010), Hg (Feyte et al. 2010; Feyte et al. 2012), Mo (Chappaz et al. 2008), and U (Chappaz et al. 2010).

This approach has been used to develop conditional equilibrium constants for the sorption of various metals (e.g., As, Cd, Cu, Ni, Pb, Zn) on diagenetic Fe and Mn oxyhydroxides (Tessier et al. 1996), which can then be used to predict metal concentrations in sediment porewater, but to our knowledge no reports have been made of direct relationships between the metal concentrations in these oxyhydroxides and metal bioavailability.

### Advantages and limitations of PSMs over other estimates of exposure

Given that freely “dissolved” metal concentrations in surface waters and sediment porewater (*C*_free_) provide a relevant exposure metric for risk assessment, PSMs must provide an accurate, reproducible estimate of the dissolved metal concentration, whether it be in the overlying water or in sediment porewater. Whether this estimate should be for total dissolved metal, total “labile” metal, or total “free” metal is discussed later. As mentioned earlier, the *C*_free_ hypothesis assumes that, for sediment-ingesting animals, the physicochemical conditions in their digestive system are similar to those in the host sediment and that porewater metal concentrations accurately reflect the chemical “activity” of the ingested sediment-bound metal.

Advantages and limitations of PSMs are outlined in the following sections. Some advantages also can be limitations, and the reverse is also possible. Thus, care must be taken in the experimental design and interpretation of results.

### Recognized advantages

- Minimal effects of sampler contamination for samplers that equilibrate with the sediment porewaters (peepers, DET)
- Improved detection limits and a simpler analytical matrix (for samplers that concentrate the contaminant, e.g., DGT)
- Time-averaged measurement during the period of deployment or equilibration
- Exclusion of particulates and colloids, and can be used with different membranes to separate dissolved metal species on the basis of molecular weight
- The DGT device can introduce selective binding layers to facilitate elemental speciation
- Episodic events and cyclic changes, which may be missed by occasional grab sampling, are incorporated into the measurement
- Reduced sampling artifacts so that measurements are more representative
- Capable of 2-dimensional measurements at high resolution, which provide exceptional insights into sediment heterogeneity and biogeochemical processes, and into metal mobilization mechanisms and
- Can be deployed in freshwater, estuarine, and marine environments, including deep sea and polar regions; in the case of DGT, the analytes accumulate in the binding resin whereas the high major ion concentration does not, which allows for lower detection limits in high-salinity matrices

### Recognized limitations

- Subject to biofouling or clogging during extended deployments, although this is less of an issue for sediment deployments because more complex biofilms do not grow because of the absence of algae
- Restricted diffusion and slow contaminant release from sediments may limit uptake rates
- Sample volumes obtained are often small
- Device design (e.g., volume sampled, size, membrane size) affects rates of metal accumulation and interpretation of the PSM response
- Interpretation of kinetic regimen passive samplers within sediments can be complex and requires appreciation of how the sediment responds (discussed later under *Models to Interpret PSM Measurements*)
- May not be representative of total bioavailability where sediment ingestion occurs
  Insertion of the sampler into the sediment can introduce oxygen and thus locally alter the redox conditions, although if the samplers are thoroughly deoxygenated and care is taken not to disturb the sediment (especially making sure the sampler is in good contact with the sediment), the impact on the measurement is minimal.

Depending on the technique employed, passive sampling may reduce or minimize the risks of sample contamination. For example, when deployed in a natural water body, any contamination initially associated with a porewater peeper or a diffusion cell will be considerably diluted during the equilibration phase. In other cases, where the PSM includes a metal-binding phase (e.g., DGT), there will be substantial preconcentration and, therefore, better detection limits.

Some PSMs (peepers and DGT particularly) are often deployed for long periods and thus yield time-averaged estimates of the ambient metal concentrations, which may be relevant in the case of long-term exposure of aquatic organisms that only slowly come to steady-state with their environment with respect to metal exposure. Long-term equilibration between the PSM and the ambient environment may underestimate the extent of short-term episodic events, but grab sampling may miss such events completely; the PSM can often detect the event, but the associated concentrations are averaged over the time of deployment (Dunn et al. 2003; Jordan et al. 2008). Allan and co-workers (2008) compared passive sampling (with DGT and Chemcatcher devices) to spot sampling, in the River Meuse (The Netherlands), and concluded “Passive sampling provides information that cannot be obtained by a realistic spot sampling frequency and this may impact on the ability to detect trends and assess monitoring data against environmental quality standards when concentrations fluctuate.” Chemcatcher samplers are typically deployed for several weeks, whereas DGT devices are often deployed for no more than 24 to 96 h (Table 1); repetitive sampling using DGT can therefore reveal temporal changes.

## TWO-DIMENSIONAL MEASUREMENTS OF METALS IN SEDIMENT

Although the potential heterogeneity of sediment has long been considered important, few techniques were actually able to observe this heterogeneity, for metals or other substances. Therefore, the investigation of metals in sediments typically involved measuring vertical concentration profiles of the solid phase or porewaters, at 1- or 2-cm resolution (Stockdale et al. 2009), despite the limitations of this approach being well known (Brendel and Luther 1995).

Toward the end of the 20th century, several techniques were described that allowed 2-dimensional measurements of porewater solutes at high resolution, which allowed sediment heterogeneity to be observed directly. Glud and co-workers described a planar optical sensor (optode) for O_2_ that used fluorescence quenching of an Ru complex and allowed very-high-resolution (100 µm), 2-dimensional measurements of O_2_ depletion across the sediment–water interface (Glud et al. 1996) and at the base of a biofilm (Glud et al. 1998). Teasdale et al. (1999) described a DGT technique for sulfide in estuarine sediment with an AgI(s) binding gel and computer-imaging densitometry, also capable of high resolution (1 mm), 2-dimensional measurements. Although these examples did not measure metals in sediment, they did provide an understanding of the sediment biogeochemical zones and heterogeneity that strongly influence metal mobilization (Stahl et al. 2012). In a crucial development, Davison et al. (1997) described 2-dimensional, high-resolution (100 µm) DGT measurements of Zn, Fe, Mn and As(III) in a microbial mat using particle-induced X-ray emission. After this, Shuttleworth et al. (1999) described an unconstrained DET device for the measurement of 2-dimensional Fe and Mn distributions in fresh water sediment porewaters at 3 × 3 mm resolution. These 2-dimensional measurements provided the advantage of measuring smaller volumes of sediment porewaters (Stockdale et al. 2009), and therefore less averaging of steep concentration gradients occurred, vertically or laterally. As a result, a more representative measurement of sediment porewater solute distributions was obtained (Pagès et al. 2011).

**Figure 4:**
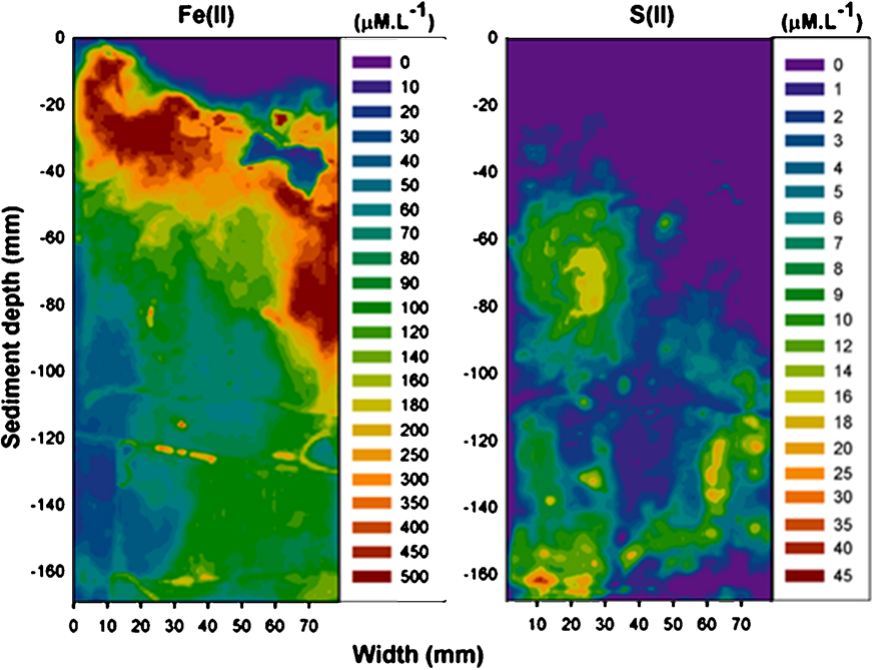
Example of 2-diomensional patterns of Fe(II) and S(II) in sediment (P. Teasdale, personal communication). The Fe(II) is a colorimetric DET measurement and the sulfide is a colorimetric DGT measurement; both measured by computer imaging densitometry at 1-mm resolution. The dark blue zone amongst the high Fe(II) at approximately 40 mm depth and 60 mm from left to right is an artifact in which the gel had broken on contact with a shell. © 2014 by the Association for the Sciences of Limnology and Oceanography, Inc.

Subsequent studies have seen the further development of the DGT technique for 2-dimensional measurements of porewater metal (As, Cu, Co, Fe, Mn, Ni, V, Zn) distributions using laser-ablation ICP-MS (Motelica-Heino et al. 2003; Warnken et al. 2004a). These approaches have allowed investigation of metal mobilization from microniches in deep sea (Fones et al. 2001) and freshwater sediment (Stockdale et al. 2008), and the mobilization of metal from sulfides (Naylor et al. 2006; 2012). Two-dimensional, high-resolution (1 mm) DET measurements of iron(II) using colorimetric reactions with ferrozine have also been described recently (Jézéquel et al. 2007; Robertson et al. 2008; Bennett, Teasdale, Welsh, Panther, Jolley et al. 2012) using low-cost computer-imaging densitometry analysis. This technique has been used to investigate the biogenic heterogeneity (Robertson et al. 2009) of coastal sediments, diurnal changes in seagrass biogeochemistry (Pagès et al. 2012), and interpret mobilization of As species from sediment (Bennett, Teasdale, Panther, Welsh, Zhao, et al. 2012). These new DGT and DET techniques provide valuable insight into the distribution of porewater metals within heterogeneous sediment, and the mechanisms of metal mobilization from microniches and attributable to changing redox conditions. These and other approaches need to be used while recognizing that biota typically tend to integrate 2-dimensional exposure patterns by actively burrowing in the sediment with subsequent modification of the microniches they reside in, for instance by irrigating their burrows with overlying water. Figure 4 shows 2-dimensional patterns of Fe(II) and S(II) obtained by a combined DGT/DET sampler (24 h deployment) in a low-density seagrass sediment on Tallebudgeera Creek, Gold Coast (Queensland, Australia); note the high degree of heterogeneity and the presence of both Fe(II), indicating a sub-oxic redox zone, and sulfide, indicating an anoxic redox zone at the same depth (50–100 mm), although at different lateral positions.

## MODELS TO INTERPRET PSM MEASUREMENTS

Early development of models to support interpretation of PSM results has helped us understand how the sediment responds to PSMs and to better understand potential sampling artifacts. All in situ porewater samplers create a diffusive flux into the sampling device on deployment (Davison et al. 2000)—the mechanism by which PSM sampling occurs—that perturbs the pseudo-equilibrium within the sediment to some extent; however, this process also occurs naturally within sediments. The response of sediment to the perturbation created by a PSM can vary considerably between equilibrium regimen (peepers and DET) and kinetic regimen (DGT) samplers, with sampler design features and sediment properties also being important.

Equilibrium samplers have a high initial flux of solutes that diminishes over time as the concentration difference driving the flux decreases. Complete equilibrium must be approached (>90%) to interpret the results as porewater concentrations. The equilibration time for such devices is determined by the ratio of the sampler volume to the exposed area of the sampler (Brandl and Hanselmann 1991). This process has been modeled (Harper et al. 1997; Davison et al. 2000) for peepers and DET PSMs, investigating 3 different regimens of solute resupply to the sediment porewaters:

i) Sustained resupply in which various processes (desorption, dissolution, microbial respiration) maintain porewater solute concentrations from the particulate phase
ii) Diffusive resupply only, in which porewater concentrations become depleted in an expanding volume from the PSM interface; and
iii) Partial resupply, which is intermediate to i) and ii) and involves both resupply from the solid phase and recharge by diffusion

Partial resupply seems to be the most common regimen encountered for trace metals (Naylor et al. 2006; Wu et al. 2011), but examples of all 3 have been observed, with the by-products of active microbial respiration most likely to be fully sustained, particularly iron(II) (Naylor et al. 2006; Zhang et al. 1995) and sulfide (Motelica-Heino et al. 2003; Robertson et al. 2009). The resupply regimen may vary considerably with depth; for instance, manganese(II) was observed to have strong resupply in surface sediments but decreased to diffusional recharge only at most other depths (Naylor et al. 2006). Equilibration time (to 95% of equilibrium) was observed to vary between 12 min and 15 h for a DET and between 23 h and 56 days for a dialysis sampler (F = 1.1 cm) in fully sustained and diffusional resupply only conditions, respectively (Davison et al. 2000). Other comparisons of models with experimental results showed that, with peepers in saline waters, equilibration times were reduced because of convection in the peeper cells, decreasing the upper deployment time (Webster et al. 1998). A 1-dimensional diffusive model was also described that allowed validation of equilibration times determined through time series deployments (Bally et al. 2005).

These resupply regimens also occur with DGT measurements, although the kinetic regimen DGT sampler maintains a higher rate of flux of analyte solutes into the sampler than do equilibrium PSMs, because of the adsorption of the analyte within the binding layer. This can be varied, however, through changing the thickness of the diffusive layer, sometimes resulting in resupply regimens also changing (Naylor et al. 2006). This option makes DGT a useful tool for investigating sediment geochemical processes (Naylor et al. 2006; 2012). Whereas DGT has the advantage that any deployment time can be used, instead of requiring uptake to have approached equilibrium, this does complicate the interpretation of the results. Under deployment conditions in which sediment interstitial water concentrations remain constant, the DGT equation can be used to calculate an accurate contaminant porewater concentration. Under all other conditions the DGT measurement is most accurately reported as a flux into the sampler (Zhang et al. 1995; Davison et al. 2000), although some authors instead report the results as concentrations and also discuss the likelihood of the porewater concentrations being underestimated (Bennett, Teasdale, Welsh, Panther, Stewart, et al. 2012). Given that the resupply can vary considerably over a concentration profile or distribution, and the difficulty of obtaining an independent measure of porewater concentrations (particularly in saline waters), this is a reasonable approach. A 2-dimensional model to assist with interpretation of DGT profiles, particularly the likely concentration and fidelity of peaks, has been developed (Harper et al. 1999; Davison et al. 2000). A method that assists in translating porewater fluxes into concentrations has also been described (Harper et al. 1999), and minimum peak width at several diffusion layer thicknesses provided for representative peak shape and concentration (Davison et al. 2000). More recently another model has been described that allows interpretation of small-scale features in DGT measurements—a spherical microniche was located close to a DGT device in the model (Sochaczewski et al. 2009). Micro-niche responses were found to most likely reflect a localized source with a higher concentration than the surrounding sediment, and most measurements of microniches are likely to reflect 62% to 87% of the true peak maxima.

Another major development toward interpretation of DGT measurements of metals in sediments was the DIFS (DGT-induced fluxes in sediments/soils) model (Harper et al. 2000; Sochaczewski et al. 2007). The DIFS allows quantitative interpretation of DGT measurements using equilibrium (K_d_) and kinetic (desorption rate) parameters that relate to conventional measurements and important geochemical processes. This response can be evaluated over time and can be used to visualize the change in sediment porewater and particulate analyte concentrations surrounding the DGT device. The DIFS has been used in soils rather than in sediment for metals because of the relative ease of making measurements on homogenized samples, which is considered relevant for soil studies. However, this approach has been used for phosphate in freshwater lake sediment (Monbet et al. 2008). A probabilistic model was developed (DGT-PROFS; Ciffroy et al. 2011) that better determines the range of physical parameters possible from a DGT measurement and was successfully evaluated against formulated sediments that varied in proportions of iron oxides (Ciffroy et al. 2011) and humic acids (Nia et al. 2011).

## LIMITATIONS: DO SPECIFIC PSMs WORK BETTER FOR SOME METALS THAN FOR OTHERS; WHICH METALS ARE (NOT) DATA-RICH?

Most studies relating PSM responses to metals in aquatic sediments or the overlying water have tended to focus on divalent species (Cd, Cu, Ni, Pb). Garmo et al. (2003) published a laboratory comparison of the behavior of 55 elements in trials with DGT devices deployed in the absence of sediments. They compared independently determined or estimated diffusion coefficients in water (i.e., *D*_H2O_, unrestricted by a gel phase) with DGT effective diffusion coefficients (*D*_DGT_) for all 55 elements. The DGTs were exposed at a controlled fluid velocity of 0.1 m · s^−1^ and a concentration of 1 µg · L^−1^ at 4 pH values between 4.7 and 6.0, and *D*_DGT_ values were determined from the uptake by the sampler. For Al, Cd, Co, Cu, Ga, Mn(II), Ni, Pb, and Zn, good agreement was seen between the measured *D*_DGT_ values and the accepted previously published *D*_H2O_ values, “with some deviations for Pb and Zn.” The uptake of Ba, Cr(III), Fe(III), Mo(VII), Sr, Ti(IV), U(IV), and V(III) proved to be pH-sensitive, and the authors reported some experimental problems that “required further investigations.” Novel *D*_DGT_ values for the lanthanides (La, Ce, Pr, Nd, Sm, Eu, Gd, Tb, Dy, Ho, Er, Tm, Tb, Yb, Lu, Y) were reported.

Accumulation of metal ions as a function of time was followed (24, 48, and 72 h) and for many of the elements linearity was observed up to 72 h (Garmo et al. 2003). Some exceptions were noted, notably for Ag(I), As(III), B(III), Bi(III), Ca, Hf, K, Li, Mg, Na, Nb, P(V), Rb, S, Sb(III), Se, Si, Sn(II), Ta, Te, Th, Tl, W, and Zr, indicating that these elements are not quantitatively collected (e.g., elements that exist in water as monovalent cations or as anions, and for which the Chelex resin does not have a high binding capacity). For such elements overall accumulation was low, and *D*_DGT_ values were less than 10% of theoretical values. Such constraints have been rectified for specific analytes, such as many of the oxyanionic species, by the incorporation of alternative adsorbents into the binding layer (e.g., metal-oxide binding phases such as ferrihydrite and titanium dioxide).

The focus of PSMs has been on individual metals, whereas sediment-dwelling biota are typically exposed to mixtures of metals of varying composition. Typically, care is taken in the deployment of the PSMs not to saturate the resins; competition for binding of individual metals to the resin usually is not a factor of importance. However, competition of metals for biotic membranes can be a factor limiting the relationship between DGT-labile metal and metal accumulated in biota (Roulier et al. 2008). Saturable biotic ligands and specific interactions between metal mixtures present in varying composition potentially complicate reliable toxicity prediction by means of PSMs.

## HOW WELL DO PSMS PREDICT BIOAVAILABILITY?

To answer the key question of how well PSMs work for metals in practice requires evidence that their response can be used to predict metal bioavailability. Ideally, such evidence would have been generated in a field setting, but such studies are very scarce in the scientific literature (Table 3, sections C and D). Accordingly, studies that were performed on natural waters or sediments but in the laboratory (e.g., algal studies on natural waters brought back to the laboratory) or in mesocosms (e.g., periphyton grown in the laboratory in mesocosms fed with natural river water; benthic organisms exposed to natural or spiked sediments) were included in a literature search regarding this question. Table 3 presents some examples in which PSMs were deployed in systems containing natural waters or sediments and where the results were compared with metal accumulation or toxicity in resident aquatic organisms.

**Table 3:** Examples of positive relationships between the response of PSMs and the response of free-living or caged aquatic organisms

PSM	Biological response	References
	A - Overlying water – fresh water	
• DGT Cu	Short-term and steady-state Cu accumulation by an aquatic moss in laboratory experiments with NOM; DGT labile Cu related to accumulation in some cases only.	(Ferreira et al. 2008)
• DGT Cu	Acute and chronic toxicity of Cu (*Ceriodaphnia dubia*) in 3 spiked river water samples containing 4 to 13 mg DOC L^−1^; the range of the LC50 values was narrower when expressed as DGT-labile Cu (20–30 µg L^−1^), but exceeded the SMAV value (11.5 µg L^−1^).	(Martin and Goldblatt 2007)
• DGT Cd	Cd uptake flux into an amphipod (*Gammarus pulex*) in laboratory studies in the presence of EDTA and Aldrich humic acid; values for [Cd]_inorg_ and [Cd]_DGT_ were very similar, indicating that few complexes were contributing to the DGT measurement.	(Pellet et al. 2009)
• DGT Al		Toxicity of Al to brown trout (fish exposed to natural waters spiked with Al and manipulated); DGT labile metal a better predictor of fish stress than total inorganic Al (pyrocatechol-violet).	(Royset et al. 2005)

B - Overlying water – seawater		
• DGT Cu	Transplanted rock oysters (*Sacccostrea glomerata*) and DGT devices were deployed over 6 weeks (biweekly measurements) in a coastal lagoon; bioaccumulated Cu strongly correlated with cumulative Cu taken up by DGT devices at a marina and a boat anchorage. These field measurements were used to calibrate the oyster biomonitors to give biomonitor-available concentrations. A significant correlation was then obtained between biomonitor-available and DGT-labile measurements over 14 sites.	(Jordan et al. 2008)
• DGT Cd Cu Ni Pb			Mussels (*Mytilus galloprovincialis*) and DGT devices were deployed together for 3 one-month periods in coastal waters of Sardinia (Italy); nonessential metals (Cd, Pb) accumulated over time and showed weak correlation with DGT-labile metal; Cu and Ni did not bioaccumulate although DGT-labile Cu and Zn were detected.	(Schintu et al. 2008)
• Hollow fiber permeable liquid membrane	Cd, Cu, Ni, and Pb uptake fluxes into a test marine alga (water from the Baltic Sea, collected at 7 different sites) as a function of [M^z+^].	(Slaveykova et al. 2009)		
• DGT Cd Cu Pb	Brown macroalga (*Padina pavonica*) collected from 5 coastal marine sites near smelters; DGT devices deployed at each site (3 d); positive correlation between DGT-labile Pb and Pb concentrations in the macroalga.	(Schintu et al. 2010)

C - Sediments – freshwater		
• Porewater peepers [Cd] in pore water in surface sediments	Cd accumulation and metallothionein induction in free-living freshwater bivalves, collected from lakes on the Canadian Shield.	(Couillard et al. 1993; Tessier et al. 1993)
• Pore water Ni	Tested 3 invertebrate taxa (*Hyalella* survival, *Gammarus* survival, and *Hexagenia* growth) in 10 Ni-spiked sediments, over at least 28 d; toxicity values based on pore water nickel concentrations had lowest among-sediment variation, especially for the 2 amphipods.	(Besser et al. 2011)
• DGT Cd			Periphyton exposed to Cd in artificial recirculating channels containing natural freshwater; Cd speciation modified by addition of nitrilotriacetate (NTA). Total and intracellular Cd content in periphyton increased and were related to an increase in DGT-labile Cd. Bioaccumulation not controlled by the free Cd concentrations, but by diffusion of labile Cd-NTA complexes.	(Bradac et al. 2009)
• DGT Cd Cu Pb	Laboratory microcosm experiments conducted with 6 contaminated sediments; compared DGT-labile metal with metal bioaccumulation in a chironomid (*Chironomus riparius*); significant relationships found for Cu and Pb; however, total metals in sediments were, surprisingly, the best predictors of bioaccumulation.	(Roulier et al. 2008)		
• DGT Cd			Laboratory microcosm experiments conducted with a natural carbonate-rich sediment spiked at 5 different concentrations; 3 test species (chironomid, *Chironomus riparius*; amphipod, *Gammarus fossarum*; mudsnail, *Potamopyrgus antipodarum*) exposed for 7 d; DGT devices also exposed 2, 4, 8, 12, 24, 48, 72, 144 h; DGT-labile Cd predicted Cd accumulation rates well in mudsnail, over-predicted rates in amphipod, and under-predicted Cd rates in chironomid.	(Dabrin et al. 2012)
• DGT Ni	Flux of Ni from spiked sediments (weeks to months) linking effects to benthic macroinvertebrate populations and *Hyalella azteca* and *Lymnea stagnalis*. No relation to benthic responses but did show biphasic flux patterns.	(Costello et al. 2011; Costello et al. 2012)		

D - Sediments—seawater		
• DGT Cu	Bioavailability of Cu to the benthic marine bivalve *Tellina deltoidalis* in sediments of varying properties contaminated with Cu-based antifouling paint particles; overlying water Cu concentrations and DGT-Cu fluxes provided good exposure concentration–response relationships in relation to Cu bioaccumulation and Cu-induced lethality.	(Simpson et al. 2012)

Provided that they are used correctly, PSMs can provide unique, relatively artifact-free concentration or flux data for sediments that cannot be obtained through grab sampling. As discussed earlier, the common PSMs for sediments are DGTs and porewater peepers, both of which are useful, but do not measure the same thing. Peepers sample either whole porewater (which may include some particulate and colloidal fractions, depending on the membrane size), whereas DGTs can measure a flux of “labile” metals. Based on the principles of the BLM (Campbell 1995; Di Toro et al. 2001), one would expect the PSMs that best predict metal bioavailability to be those that respond only to the free metal ion, or (failing that) respond only to inorganic metal (i.e., the free metal ion and its inorganic complexes), such that one can calculate the free metal ion concentration confidently using the inorganic metal concentration, the inorganic ligand concentrations, pH, and ionic strength as inputs to an equilibrium speciation model. PSMs that collect “labile” metal are inherently less likely to be useful in this context, because the nature of what is included in the labile fraction (e.g., weak metal complexes) will vary from one receiving water to another. For example, Buzier et al. (2006) studied the bioavailability of Cd and Cu in wastewaters and showed that DGT-labile Cd overestimated bioavailability to *Daphnia magna* (see their Figure 1). Similarly, Ferreira et al. (2008) reported that DGT-labile Cu was a good predictor of Cu bioaccumulation in moss in the presence of some types of organic matter but not others. Conversely, using Aldrich humic acid (high molecular weight) and ethylenediaminetetra-acetic acid, Pellet et al. (2009) showed that [Cd]_DGT_ and [Cd]_inorg_ were very similar and that the predictive powers of the 2 variables were accordingly also very similar.

Metal bioavailability also depends on the aquatic species of interest. For example, Dabrin et al. (2012) conducted laboratory microcosm experiments with a natural carbonate-rich sediment spiked with Cd at 5 different concentrations. Three different test species (chironomid, *Chironomus riparius*; amphipod, *Gammarus fossarum*; mudsnail, *Potamopyrgus antipodarum*) were exposed for 7 d to the spiked sediments. The DGT-labile Cd predicted Cd accumulation rates well in the mudsnail, overpredicted Cd accumulation rates in the amphipod, and underpredicted Cd accumulation rates in the chironomid.

In a recent study (Costello et al. 2012), sediments were amended with Ni and placed either within a streamside mesocosm or deployed in situ. Using macroinvertebrate community composition as a response, the authors compared the performance of DGT-measured Ni with AVS and organic carbon–corrected Ni ((SEMNi-AVS)/f_OC_) and total Ni concentrations as predictors of the biological response. From both experiments, DGT-labile Ni poorly predicted the invertebrate response to metal, whereas models that included total Ni or (SEMNi-AVS)/f_OC_ predicted the invertebrate response for the streamside mesocosm and in situ experiments, respectively. The authors concluded that DGT overestimated the available Ni fraction, possibly because the DGT resin may be mobilizing a solid-phase Ni fraction that is not bioavailable to the colonizing benthic invertebrates, or because the binding of dissolved Ni to the DGT is not affected by other dissolved cations that may be protecting the invertebrates (Costello et al. 2012).

In a similar study (Simpson et al. 2012), a Cu-sensitive estuarine bivalve (*Tellina deltoidalis*) was exposed to 3 sediments with varying properties that had been artificially contaminated with Cu-based antifouling paint particles. Overlying water Cu concentrations and DGT-Cu fluxes measured at the sediment–water interface provided good exposure concentration–response relationships relative to Cu bioaccumulation and Cu-induced lethality.

In summary, and as illustrated in Table 3, an encouraging correspondence between metal bioavailability and PSM-measured metal has been demonstrated for the water column. However, very few studies have deployed PSM devices in metal-contaminated sediments and then compared the PSM response to metal accumulation or metal toxicity in benthic organisms; the results from these sediment studies are less persuasive than those obtained for the water column. This situation may simply reflect the paucity of sediment studies, but it should also be recalled that PSMs respond to aqueous metals and thus can only indirectly account for the uptake of diet-borne metals. Clearly the correspondence between bioavailability and PSM-measured metal for sediment-dwelling organisms remains to be confirmed. Nevertheless, the geochemical information furnished by PSMs is clearly “complementary” and can be used to help interpret the results of metal bioaccumulation assays or toxicity tests conducted on sediments. Understanding the fraction sampled by a PSM will be critical for assessing biological routes of exposure (e.g., dissolved vs colloidal vs particulate-associated metals) and any resulting effects.

## BARRIERS TO WIDESPREAD APPLICATION OF PSMs BY DECISION MAKERS

A list of barriers to broader regulatory acceptance of PSMs was articulated by participants in a recent workshop on research and development needs for the long-term management of contaminated sediments (Thompson et al. 2012); one of the principal barriers identified was that too few studies relate PSM responses to actual metal-induced effects in benthic organisms.

In a regulatory context, data from PSMs are more readily accepted by decision makers where unambiguous linkage to parameters has been established and vetted by the scientific community. Examples are parameters such as total dissolved metal concentration (which could then be used as input to a chemical equilibrium model and the BLM, or as partial input to a biodynamic model), or the free metal ion concentration. Such information is consistent with the recognition in current regulations that metal speciation is critical to risk assessment and that the free metal ion concentration, as opposed to the total concentration, is a useful predictor of metal bioavailability. However, techniques that express results in a manner that is less directly linked to regulatory paradigms, for example, that measure the rate of metal accumulation (such as the DGT), will likely meet with more resistance. In such cases one must back-calculate the concentration that would yield the observed metal flux. This is straightforward for synthetic solutions and controlled conditions in the laboratory, but more complex for field deployments in which empirical measurements of metal flux are affected by a variety of physicochemical factors, including turbulence, temperature, water chemistry (i.e., pH; hardness, and dissolved organic carbon), and the potential of biofouling of the passive sampler. Despite these limitations, PSMs allow for a much more representative exposure measurement than do grab samples and subsequent determination of total porewater concentrations. The PSMs are highly complementary and as useful as or more useful than conventional sediment porewater measurements.

## CONCLUSIONS AND RECOMMENDATIONS

### Conclusions

- The PSMs have been useful for evaluating the geochemical behavior of metals in surficial sediments, including the determination of fluxes across the sediment–water interface, and postdepositional changes in metal speciation and/or forms (diagenesis). These studies have indicated a marked geochemical heterogeneity in sediments, at the scale of millimeters to micrometers.
- The development of PSM techniques capable of measuring 2-dimensional, high-resolution (mm–100 µm) porewater solute distributions suggests that conventional measurements of porewaters in sediments, such as filtering, squeezing, or centrifuging sediments to separate the interstitial water from the solid phase, may lead to major artifacts. The PSMs can also characterize fine-scale changes across the sediment–water interface better than conventional measurements.
- The PSMs have been less useful for evaluating the bioavailability of metals in surficial sediments with respect to benthic organisms. Representing diverse and often complex biouptake mechanisms with simple chemical processes remains a challenge, even though PSMs can operate in different regimens (equilibrium or kinetic).
- Equilibrium devices such as porewater “peepers” show promise for those benthic organisms that are exposed to metals primarily through contact with the porewater. Using the overall composition of the porewater, one may apply modeling (e.g., the BLM) to estimate metal bioavailability in porewater, similar to what has been done for the overlying water.
- Non-equilibrium devices designed to measure metal fluxes (e.g., DGT) are often cited in the literature as tools for quantifying metal bioavailability, but very few examples were identified in which such measures have yielded significant improvement over conventional analysis of the sediment. This result contrasts with the situation for metals in the soil environment, where the DGT approach has been shown to improve predictions of metal bioavailability. In soils, the supply of metals from the solid phase and their physical transport from the solid phase to the biological interface (e.g., root surface or invertebrate epithelium) are known to influence metal uptake; under such conditions, the use of a PSM to measure metal fluxes makes sense. In contrast, for sediments in which porewater serves as a contiguous medium between the solid phase and the biological target, and where equilibrium between solid phase and porewater is less likely perturbed by biological uptake, metal flux measurements may be less relevant than free metal concentrations.
- The DGT technique has the most potential for versatility. New binding layers could be used that have lower binding capacities and selectivity for metals that may better mimic the saturable kinetics often observed in biouptake and related biotic ligands that are the sites of toxic action (i.e., between the kinetic and equilibrium regimes traditionally used by PSMs).

### Recommendations

To optimize PSM use for metals and promote sound interpretation of PSM data in risk assessment approaches that allow for assessment of metal bioavailability in sediment, further work is needed:

- Standardization of devices is clearly needed. For broad acceptance of PSMs, round-robin testing should be performed to determine intra-versus interlaboratory variance. Such efforts will be facilitated by standardization of sample and sampler preparation, and the creation of reference materials.
- For each PSM, clear guidance on methods and sampling protocols, and on calibration of devices, are also needed, including noting any limitations on the types of sediment that can be conveniently measured.
- Very few studies have tried to link PSM responses in sediments to metal uptake and toxicity responses in benthic organisms. Further studies of this type are needed.
- Future PSMs could be designed to mimic saturable kinetics, which would fill the gap between the kinetic and the equilibrium regimen samplers currently used, and may represent the accumulation of metals by benthic organisms more accurately.
- The few studies relating PSM responses to metal bioavailability in the water column or in aquatic sediments have tended to focus on Cd, Cu, Ni, and Pb; on a relative scale, these metals are “data-rich.” Future work on linking the bioavailability of data-poor metals and metalloids (e.g., Hg, Se) to PSM responses is required to expand applicability across sites.
- The capability of DGT PSMs to selectively measure the different oxidation states of arsenic in situ, at high resolution and in 2 dimensions, is a significant advantage of these techniques over conventional sediment sampling approaches. Because these methods are relatively new, currently no published studies exist using these devices to investigate arsenic toxicity and bioavailability in sediments.
- The PSM measurements should be linked with existing and future models, including those that define metal speciation under equilibrium conditions. When comparing the performance of different PSMs, care should be taken to obtain complementary water and sediment chemistry data, so that *C*_free_ can be calculated. The comparison of PSM data and modeled speciation data is a powerful means of evaluating the performance of both the passive sampling device and the chemical equilibrium model.
- Presently, the application of such models often results in predictions of metal speciation that diverge from the results of measurements with PSMs. One of the research priorities should be to work to improve the modeled predictions of metal speciation, so that they can be used to derive free metal ion concentrations from the time-averaged concentrations obtained from PSMs. In turn, the time-averaged free metal ion concentrations could then be used in BLM calculations.
- Furthermore, the use of well-developed PSMs for routine monitoring alongside conventional approaches needs to be undertaken.

## Supporting Information

All Supplemental Data may be found in the online version of this article.

Case studies on the application of PSMs for investigating metals and metalloids in sediments.

Figure S1 SI.1. Application of DGT to mercury and methyl mercury availability in sediments SI.2. PSMs for investigating arsenic in sediments Figures S1.1-3
