# Supplementary material for: Passive sampling methods for contaminated sediments: State of the science for metals
Source: Integr Environ Assess Manag. 2014 Jan 27;10(2):179–96. doi: 10.1002/ieam.1502 (PMC4238822; doi:10.1002/ieam.1502)
Supplement: Figure S1 — SI.1. Application of DGT to mercury and methyl mercury availability in sediments SI.2. PSMs for investigating arsenic in sediments Figures S1.1-3 [file ieam0010-0179-SD1.doc]

**Passive Sampling Methods for Contaminated Sediments: State of the Science for Metals**

**Supplementary Information**

Running head: PSMs for metals in assessment of contaminated sediments

Willie J.G.M. Peijnenburg*^,†,‡^, Peter R. Teasdale^§^, Danny Reible^||^, Julie Mondon^#^, William W. Bennett^§^, Peter G.C. Campbell^††^

†Institute of Environmental Sciences (CML), University of Leiden, Leiden, The Netherlands. [peijnenburg@cml.leidenuniv.nl](mailto:peijnenburg@cml.leidenuniv.nl)

‡National Institute for Public Health and the Environment, Center for Safety of Substances and Products, PO Box 1, 3720 Bilthoven, The Netherlands. [willie.peijnenburg@rivm.nl](mailto:willie.peijnenburg@rivm.nl)

^§^Environmental Futures Centre, School of Environment, Griffith University, Gold Coast Campus, Australia. [p.teasdale@griffith.edu.au](mailto:p.teasdale@griffith.edu.au), [w.bennett@griffith.edu.au](mailto:w.bennett@griffith.edu.au)

^||^Center for Research in Water Resources, University of Texas, Austin, TX 78758 USA. [reible@mail.utexas.edu](mailto:reible@mail.utexas.edu)

^#^Center for Integrated Ecology, Environmental Sustainability Research Cluster, Deakin University, Warrnambool Campus, PO Box 423, Warrnambool Victoria 3280 Australia. [julie.mondon@deakin.edu.au](mailto:julie.mondon@deakin.edu.au)

^††^Université du Québec, Institut national de la recherche scientifique, Centre Eau, Terre et Environnement, 490 de la Couronne, Québec, QC, Canada G1K 9A9. [peter.campbell@ete.inrs.ca](mailto:peter.campbell@ete.inrs.ca)

*To whom correspondence may be addressed: National Institute for Public Health and the Environment, PO Box 1, 3720 Bilthoven, The Netherlands. T: 0031-30-2743129, E: [willie.peijnenburg@rivm.nl](mailto:willie.peijnenburg@rivm.nl)

**CASE STUDIES ON THE APPLICATION OF PSMs FOR INVESTIGATING METALS AND METALLOIDS IN SEDIMENTS**

***SI.1. Application of DGT to mercury and methyl mercury availability in sediments***

The DGT-approach has only recently been applied to the practical measurement of mercury in sediment pore water. The goal of monitoring mercury in sediment pore water is to define the contaminant available for migration to the surface of the sediments and overlying water and to define the mercury concentration available for methylation. Total and methyl mercury (total and MeHg) in sediment pore water should indicate the mobile concentrations of these species and the free mercury concentrations in pore water are likely to be better linked to methylation than bulk solid phase mercury. Methylated mercury is the form of mercury of most concern and the dominant form that bioaccumulates in higher organisms. The presumed relationship between DGT-measured mercury and bioavailable mercury assumes that mercury precipitated into a solid phase, such as the low solubility cinnabar (HgS), is not mobile or available for methylation. Methyl mercury forms when certain specific conditions are met, typically :

- Presence of free mercury (as opposed to sorbed mercury);
- Presence of mercury sulfur complexes in a bioavailable form; and,
- Ongoing reduction, particularly sulfate reduction.

Mercury sulfur complexes that are bioavailable were once considered limited to dissolved species although recent work has suggested that the mercury sulfide may be in a nanoparticulate form. The DGT approach to sample pore waters provides the opportunity to measure the concentration of mercury in both dissolved and fine nanoparticulate form. As particle sizes grow in the tens of nanometer ranges, however, the diffusion of such particles (more than 1000 times lower than dissolved species diffusion coefficients) becomes too slow to contribute appreciably to uptake in the sorbing resin. The relationship of DGT measurements of mercury to biologically relevant mercury has been demonstrated in the overlying water but not in sediments; more research is required to provide confirmation. Such research should relate bioaccumulation of mercury in benthic organisms or rates of mercury methylation to DGT measured mercury concentrations. Methyl mercury is less hydrophobic than inorganic mercury species and thus DGT measurements of mercury are likely to be dominated by dissolved methyl mercury . The DGT measurements of methyl mercury could be used to estimate mercury methylation rates and the concentration of available and mobile methyl mercury.

Different materials are required to apply DGT for mercury and methyl mercury due to limited sorption of the typically employed resins for mercury species and potential interactions within the diffusion layer. Diviš et al. found that the Chelex®-100 resin does not demonstrate a high affinity for mercury ions and recommended a Spheron-Thiol based resin. The resin, unfortunately, is no longer available and Clarisse and Hintelmann demonstrated that a 3-mercaptopropyl-functionalized silica gel based resin could be used within DGT to measure both total Hg and MeHg in solution. The sorption capacity of the 3-mercaptopropyl functionalized silica gel is large, and nearly complete extraction of the mercury and methyl mercury after exposure is possible . The diffusive gel employed is also normally based on an agarose solution, instead of the traditional polyacrylamide solution due to the affinity of mercury ions for the amine groups in polyacrylamide .

The application of the DGT device, once constructed to meet the needs of mercury and methyl mercury measurement, is traightforward. The DGT exposed surface (i.e., a filter layer covering the diffusion layer) is exposed to the contaminated sediment for a period of time. Initially the uptake of Hg and MeHg into the sorbing resin is linear and directly proportional to the concentration in the adjacent pore waters. Eventually (typically after 4-10 days) , the pore water around the device can be depleted of mercury and uptake is no longer at a constant rate over time but instead decreases over time, which makes it more difficult to analyze and interpret longer exposures .

***SI.2. PSMs for investigating arsenic in sediments***

The geochemical behavior of arsenic in sediments has been the subject of comprehensive research in recent years, stemming primarily from the importance of mitigating the human health consequences of naturally mobilized arsenic in groundwater consumed by millions of people in Bangladesh and India. Despite this large research effort, however, uncertainties still remain with regard to the mechanistic processes associated with the mobilization of arsenic in sediment systems. Understanding these processes, and being able to investigate them *in situ*, is essential for studying the bioavailability and toxicity of arsenic in sediment systems.

Peepers have been successfully used to investigate arsenic geochemistry in a variety of aquatic systems and have provided important data of higher quality than traditional core slicing and extraction techniques. Unfortunately, peepers still suffer from the same difficulties as conventional porewater sampling techniques, in that it can be difficult to maintain the speciation of arsenic when they are removed from the sediment for transport, storage, and processing. This issue has been solved by the use of PSMs such as the DGT, which accumulate arsenic species *in situ* and immobilize them onto a selective binding phase.

The DGT technique has been developed to allow the measurement of total inorganic arsenic using either a ferrihydrite binding phase or a Metsorb (TiO_2_) binding phase . Both techniques have undergone extensive method validation and perform equally well in freshwater systems . In marine systems, however, ferrihydrite DGT experiences competition effects from the major ions in solution and tends to underestimate dissolved arsenic concentrations, whereas Metsorb DGT performs accurately in both fresh and marine environments . An additional DGT technique, described by Bennett and co-workers , utilizes a thiol-based adsorbent (3-mercaptopropyl functionalized silica gel) that is highly selective for the reduced species of arsenic (arsenite) in both fresh and marine systems. In waters, these DGT techniques can be deployed concurrently to allow measurement of inorganic arsenic speciation; arsenite is measured selectively by a 3-mercaptopropyl functionalized silica DGT, total inorganic arsenic is measured by Metsorb or ferrihydrite DGT, and arsenate is calculated by difference .

DGT techniques have been successfully applied to investigating arsenic sediment geochemistry and have provided new insight into both the fine-scale spatial distribution and mobilization of arsenic. Stockdale and co-workers demonstrated the high-resolution measurement of arsenic in a freshwater sediment using a ferrihydrite DGT that was analyzed at a resolution of 333 μm by laser ablation–ICP-MS. This gave evidence of significant spatial heterogeneity in the form of microniches, where analyte concentrations varied considerably over sub-millimeter spatial scales (see also the section on Two-dimensional measurements of metals in sediment).

Bennett and co-workers investigated arsenic mobilization processes in freshwater, estuarine water, and marine sediments using Metsorb DGT and colorimetric Fe(II) DET techniques to examine the relationship between the reductive dissolution of iron oxide minerals and arsenic release to the pore water. These techniques showed a strong positive relationship between mobilization of total As and Fe(II) in sediment pore waters at high resolution (1-3 mm). This work was expanded to show the effect of water-column anoxia on arsenic mobilization in fresh and marine sediments. In that study, DGT samplers with 3-mercaptopropyl functionalized silica gel binding phases were deployed alongside Metsorb DGT samplers to obtain high-resolution arsenic speciation information. As(III) was the primary mobilized species during reductive dissolution of iron oxide minerals for both fresh and marine sediments. This has important implications for the toxicity assessment of arsenic in sediments, as the oxidation state of arsenic strongly influences the bioavailability and toxicity of arsenic to biota .

The only study to date that has utilized DGT to investigate arsenic toxicity involved wheat grown in arsenic contaminated soils. Mojsilovic and co-workers used ferrihydrite DGT to measure arsenic to phosphate ratios in the pore water of various contaminated soils, which were compared with wheat biomass as the response variable. They found that the DGT-measured arsenic to phosphate ratio was a strong predictor of wheat toxicity (EC50 of 0.246 with 95% CI of 0.168-0.360; R^2^ = 0.893).

The capability of DGT PSMs to selectively measure the different oxidation states of arsenic *in situ*, at high resolution and in two dimensions, is a significant advantage of these techniques over conventional sediment sampling approaches. Since these methods are relatively new, there are currently no published studies using these devices to investigate arsenic toxicity and bioavailability in sediments.

***References***

Andrade CF, Jamieson HE, Kyser TK, Praharaj T, Fortin D. 2010. Biogeochemical redox cycling of arsenic in mine-impacted lake sediments and co-existing pore waters near Giant Mine, Yellowknife Bay, Canada. Applied Geochemistry 25(2):199-211.

Belzile N, Tessier A. 1990. Interactions between arsenic and iron oxyhydroxides in lacustrine sediments. Geochimica et Cosmochimica Acta 54:103-109.

Bennett WW, Teasdale PR, Panther JG, Welsh DT, Jolley DF. 2010. New diffusive gradients in a thin film technique for measuring inorganic arsenic and selenium(IV) using a titanium dioxide based adsorbent. Analytical Chemistry 82(17):7401-7407.

Bennett WW, Teasdale PR, Panther JG, Welsh DT, Jolley DF. 2011. Speciation of dissolved inorganic arsenic by diffusive gradients in thin films: selective binding of As-III by 3-mercaptopropyl-functionalized silica gel. Analytical Chemistry 83(21):8293-8299.

Bennett WW, Teasdale PR, Panther JG, Welsh DT, Zhao HJ, Jolley DF. 2012a. Investigating arsenic speciation and mobilization in sediments with DGT and DET: A mesocosm evaluation of oxic-anoxic transitions. Environmental Science & Technology 46(7):3981-3989.

Bennett WW, Teasdale PR, Welsh DT, Panther JG, Stewart RR, Price HL, Jolley DF. 2012b. Inorganic arsenic and iron(II) distributions in sediment porewaters investigated by a combined DGT-colourimetric DET technique. Environmental Chemistry 9(1):31-40.

Benoit JM, Gilmour CC, Mason RP, Heyes A. 1999a. Sulfide controls on mercury speciation and bioavailability to methylating bacteria in sediment pore waters. Environmental Science and Technology 33:951-957.

Benoit JM, Mason RP, Gilmour CC. 1999b. Estimation of mercury-sulfide speciation in sediment pore waters using octanol-water partitioning and implications for availability to methylating bacteria. Environmental Toxicology and Chemistry 18(10):2138-2141.

Chess TW. 2010. Laboratory optimization and field demonstration of diffusive gradients in thin films for in-situ mercury measurements in river sediments. [Austin, TX, USA]: University of Texas. p. 78.

Clarisse O, Dimock B, Hintelmann H, Best EPH. 2011. Predicting net mercury methylation in sediments using diffusive gradient in thin films measurements. Environmental Science & Technology 45(4):1506-1512.

Clarisse O, Hintelmann H. 2006. Measurements of dissolved methylmercury in natural waters using diffusive gradients in thin film (DGT). Journal of Environmental Monitoring 8(12):1242-1247.

Clarisse O, Lotufo GR, Hintelmann H, Best EPH. 2012. Biomonitoring and assessment of monomethylmercury exposure in aqueous systems using the DGT technique. Science of the Total Environment 416:449-454.

Deonarine A, Hsu-Kim H. 2009. Precipitation of mercuric sulfide nanoparticles in NOM-containing water: implications for the natural environment. Environmental Science & Technology 43(7):2368-2373.

Diviš P, Leermakers M, Docekalova H, Gao Y. 2005. Mercury depth profiles in river and marine sediments measured by the diffusive gradients in thin films technique with two different specific resins. Analytical and Bioanalytical Chemistry 382(7):1715-1719.

Diviš P, Szkandera R, Brulik L, Docekalova H, Matus P, Bujdos M. 2009. Application of new resin gels for measuring mercury by diffusive gradients in a thin-films technique. Analytical Sciences 25(4):575-578.

Gerbig CA, Kim CS, Stegemeier JP, Ryan JN, Aiken GR. 2011. Formation of nanocolloidal metacinnabar in mercury-DOM-sulfide systems. Environmental Science & Technology 45(21):9180-9187.

Jain CK, Ali I. 2000. Arsenic: Occurrence, toxicity and speciation techniques. Water Research 34(17):4304-4312.

Johnston SG, Keene AF, Burton ED, Bush RT, Sullivan LA, McElnea AE, Ahern CR, Smith CD, Powell B, Hocking RK. 2010. Arsenic mobilization in a seawater inundated acid sulfate soil. Environmental Science & Technology 44(6):1968-1973.

Lehto NJ, Davison W, Zhang H, Tych W. 2006. An evaluation of DGT performance using a dynamic numerical model. Environmental Science & Technology 40(20):6368-6376.

Martin AJ, Pedersen TF. 2004. Alteration to lake trophic status as a means to control arsenic mobility in a mine-impacted lake. Water Research 38(20):4415-4423.

Mason RP, Reinfelder JR, Morel FMM. 1996. Uptake, toxicity, and trophic transfer of mercury in a coastal diatom. Environmental Science and Technology 30:1835-1845.

Mojsilovic O, McLaren RG, Condron LM. 2011. Modelling arsenic toxicity in wheat: Simultaneous application of diffusive gradients in thin films to arsenic and phosphorus in soil. Environmental Pollution 159(10):2996-3002.

Panther JG, Stillwell KP, Powell KJ, Downard AJ. 2008. Development and application of the diffusive gradients in thin films technique for the measurement of total dissolved inorganic arsenic in waters. Analytica Chimica Acta 622(1-2):133-142.

Stockdale A, Davison W, Zhang H. 2008. High-resolution two-dimensional quantitative analysis of phosphorus, vanadium and arsenic, and qualitative analysis of sulfide, in a freshwater sediment. Environmental Chemistry 5(2):143-149.

**Figure SI.1.** A typical porewater peeper design (modified from ). Other styles of peepers are illustrated in .


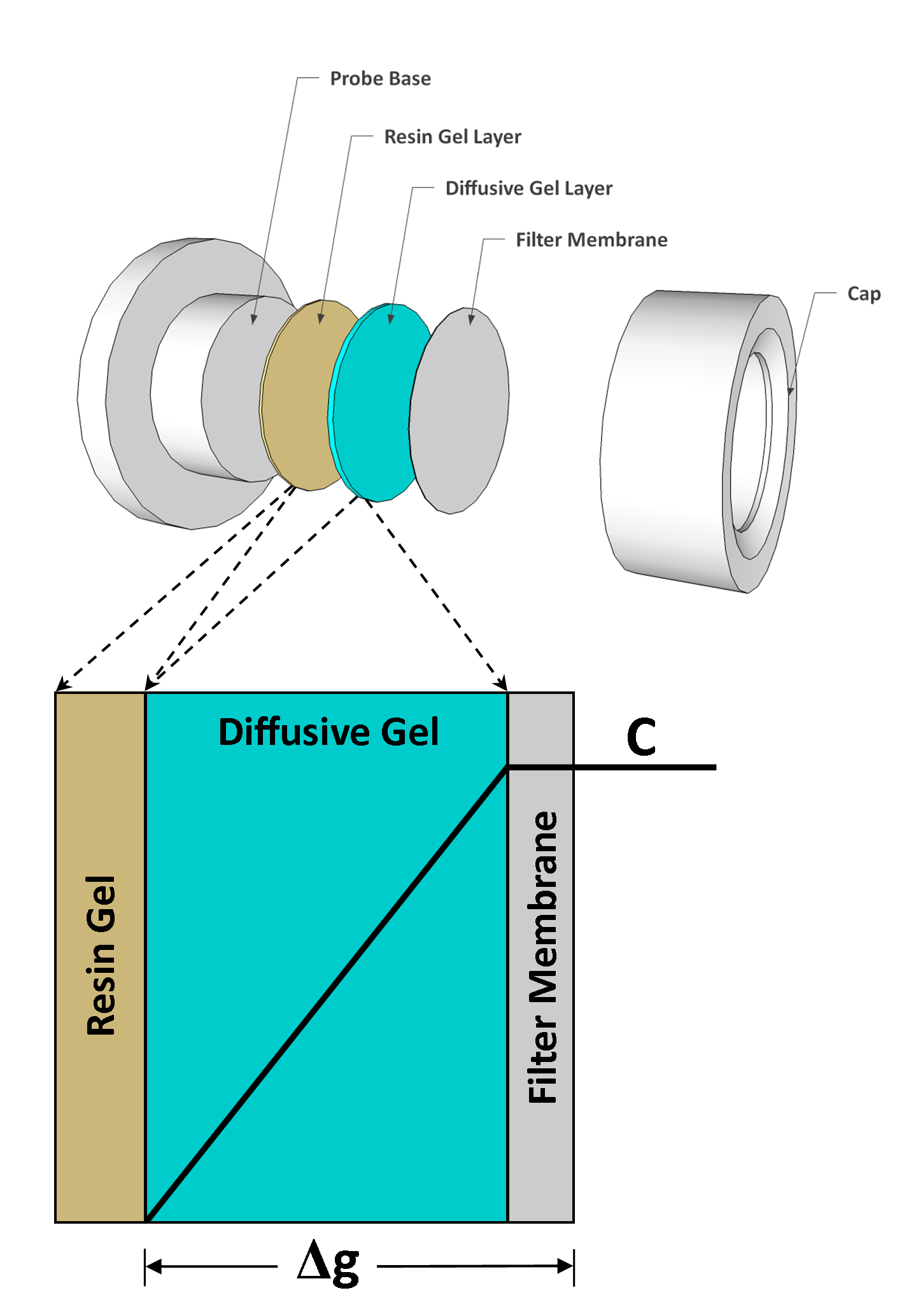


**Figure SI.2.** Illustration of DGT operation (modified from ). C is the concentration of the external waters. Δg is the thickness of the diffusive layer path (including the membrane).


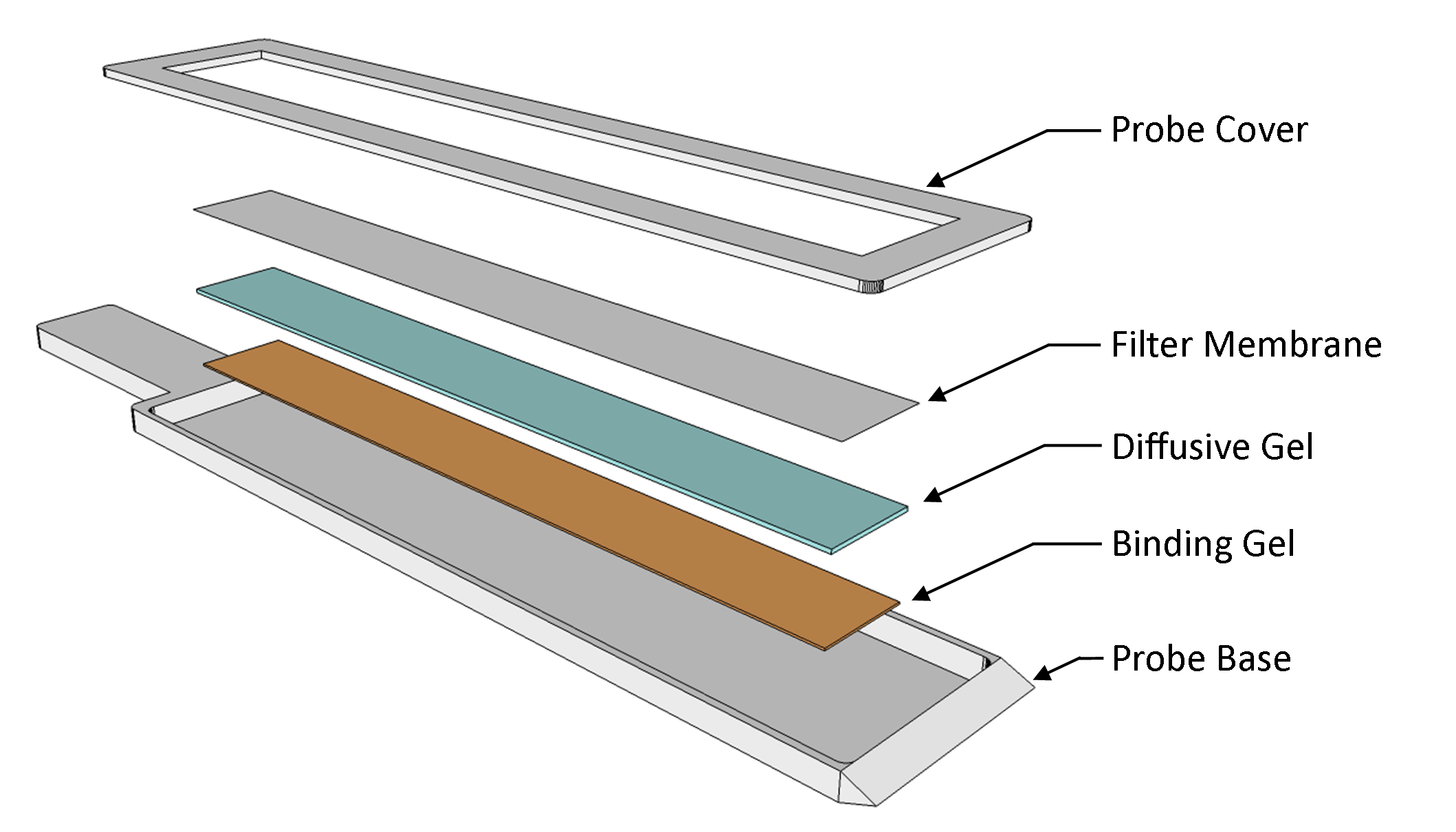


**Figure SI.3.** Configuration of the DGT device use for sediment deployments (modified from ). This device is also used for unconstrained DET deployments (without inclusion of a binding gel).
